# Ginsenoside Rb1 mitigates atherosclerosis in part through modulating FTO-mediated m^6^A RNA modification in NETs-induced endothelial activation

**DOI:** 10.3389/fphar.2025.1631076

**Published:** 2025-07-23

**Authors:** Zhenni Yang, Minqi Xiong, Xinmiao Tang, Peiwei Wang, Jingang Cui, Yu Chen, Teng Zhang

**Affiliations:** ^1^Yueyang Hospital of Integrated Traditional Chinese and Western Medicine, Shanghai University of Traditional Chinese Medicine, Shanghai, China; ^2^Clinical Research Institute of Integrative Medicine, Shanghai Academy of Traditional Chinese Medicine, Shanghai, China; ^3^Laboratory of Clinical and Molecular Pharmacology, Yueyang Hospital of Integrated Traditional Chinese and Western Medicine, Shanghai University of Traditional Chinese Medicine, Shanghai, China

**Keywords:** Ginsenoside Rb1, atherosclerosis, endothelial activation, neutrophil extracellular traps, fat mass and obesity-associated protein

## Abstract

**Background:**

Ginsenoside Rb1 (Rb1) exerts pharmacological effects in attenuating the progression of atherosclerosis. However, whether the anti-atherosclerotic effects of Rb1 involve suppressing neutrophil extracellular traps (NETs)-induced endothelial activation and whether N^6^-methyadenosine (m^6^A) RNA modification is mechanistically implicated in this process remain unknown.

**Methods:**

High fat diet (HFD)-fed *Apoe* ^−/−^ mice were subjected to histological, immunohistological and molecular biological analyses. Moreover, NETs-induced endothelial activation and m^6^A RNA methylation were assessed in human aortic endothelial cells (HAECs).

**Results:**

Rb1 mitigated atherosclerotic lesions and endothelial activation *in vivo*. Rb1 diminished adhesion of neutrophils and monocytes to NETs-stimulated HAECs, offset NETs-upregulated endothelial expression of *ICAM1*, *VCAM1*, *SELE* and *SELP*, and counteracted NETs-induced endothelial barrier impairment *in vitro*. NETs exposure significantly decreased the level of m^6^A RNA methylation and increased the expression of demethylase fat mass and obesity-associated protein (FTO) in HAECs, whereas Rb1 treatment enhanced m^6^A RNA methylation and reduced FTO expression in the NETs-stimulated HAECs. Overexpression of *FTO* abrogated the protective effects of Rb1 against NETs-induced endothelial activation in HAECs. Furthermore, *Fto* overexpression in endothelial cells partially abolished Rb1-confered attenuation of atherosclerotic pathologies and the aortic expression of Vcam1 in HFD-fed *Apoe* ^−/−^ mice.

**Conclusion:**

The work here demonstrates that Rb1 mitigates atherosclerosis in part by suppressing NETs-induced endothelial activation. Mechanistically, the pharmacological effects of Rb1 in attenuating NETs-induced endothelial activation are in part mediated by modulating FTO-mediated m^6^A RNA demethylation in endothelial cells.

## 1 Introduction

Atherosclerosis is the major pathological basis of cardiovascular disorders. Mechanistically, disruption of the functional integrity of the arterial endothelium proceeds aberrant interplay between leukocytes and activated endothelial cells, which initiates a cascade of events leading to formation of atherosclerotic plaques (Gimbrone et al., 2016; Boulanger, 2016). Thus, the pivotal roles of the activated endothelium in coordinating the inflammatory responses occurring in the arterial walls call for therapies targeting endothelial activation, which holds potential for improving the clinical outcome in atherosclerosis management.

The pro-atherogenic roles of abnormally activated neutrophils have been gaining increased attention. Neutrophils are the predominant leukocytes that roll along the endothelium during atherosclerosis (Eriksson et al., 2001). The neutrophil presence is consistently noted in atherosclerotic lesions (Trillo, 1982; van Leeuwen et al., 2008; Yvan-Charvet et al., 2008; Drechsler et al., 2010). Available evidence also supports that neutrophil extracellular traps (NETs) released from activated neutrophils promote atherogenesis (Josefs et al., 2020; Knight et al., 2014). NETs primarily contain extracellularly released decondensed chromatin and participate in neutrophils’ orthodox function of bacterial killing (Brinkmann et al., 2004). Accumulating evidence underscores the important implications of NETs in sterile inflammation that contributes to the pathophysiological changes of a broad range of non-infectious disorders including atherosclerosis (Knight et al., 2014; Döring et al., 2012; Dörin et al., 2012; Warnatsch et al., 2015; Jorch and Kubes, 2017). Of note, NETs can cause direct damage to the endothelial cells (Gupta et al., 2010). Therefore, alleviating NETs-induced endothelial damage is of therapeutic potential in mitigating atherosclerosis.

N^6^-methyadenosine (m^6^A) RNA modification exerts crucial impact on RNA metabolism, thereby modulating fundamental cellular processes by regulating expression levels of multiple functionally related gene targets (He and He, 2021). Decreased level of m^6^A RNA modification has been noted in both early phase and advanced atherosclerotic plaques (Quiles-Jiménez et al., 2020). Overexpression of the gene encoding fat mass and obesity-associated protein (FTO), a demethylase that reverses m^6^A RNA modification, promotes endothelial activation; whereas *FTO* knockdown mitigates endothelial activation (Mo et al., 2022). Thus, the mechanistic implications of FTO-mediated m^6^A RNA demethylation in endothelial activation offer a new angle to explore the pharmacological mechanisms of potential anti-atherosclerotic therapies that modulate endothelial activation.

*Panax ginseng* C.A.Mey. Has a broad application in the clinical management of atherosclerotic cardiovascular diseases (Kim, 2012). Ginsenosides, the major metabolites of *P. ginseng* C.A.Mey., are equipped with anti-atherosclerotic effects (Duan et al., 2017; Fan et al., 2012). Ginsenoside Rb1 (Rb1), one of the most abundant ginsenosides, attenuates atherosclerosis through inhibiting proinflammatory activation of macrophages and endothelial cells (Zhou et al., 2018; Qiao et al., 2017; Zhang et al., 2018; Zhou et al., 2017). However, whether the anti-atherosclerotic effects of Rb1 implicate mitigating NETs-induced endothelial activation is unclear. Additionally, it remains unknown whether Rb1 is pharmacologically active at modulating FTO-mediated m^6^A demethylation in endothelial activation in the presence of NETs. To address these questions, we evaluated the pharmacological impact of Rb1 on NETs-induced endothelial activation in the pathological context of atherosclerosis. Furthermore, we also investigated FTO-mediated m^6^A RNA demethylation to understand the mechanisms contributing to the pharmacological effects of Rb1 against NETs-induced endothelial activation in atherosclerosis.

## 2 Materials and methods

### 2.1 Reagents

Rb1 (purity >98.0%, B21050) was obtained from Shanghai Yuanye Biotechnology Co., Ltd. (China). Endothelial cell culture medium (ECM) was product from ScienCell Research Laboratories (United States). RPMI1640 medium and Opti-MEM™ I reduced serum medium were products from Thermo Fisher Scientific (United States). Evans blue dye was ordered from Solarbio (China). Phorbol 12-myristate 13-acetate (PMA) was ordered from Sigma-Aldrich (United States).

### 2.2 Animals and treatments

*Apoe* ^−/−^ male mice (C57BL/6J background) were supplied by the Vital River Laboratory Animal Technology Co., Ltd. (Beijing, China). The animals were accommodated in standardized laboratory conditions in the Animal Resource Center at Yueyang Hospital of Integrated Traditional Chinese and Western Medicine. For experiments without *Fto* overexpression in endothelial cells, *Apoe* ^−/−^ mice (8-week old) on the diet containing 20% fat and 1.5% cholesterol (high fat diet, HFD) (Shanghai SLAC Laboratory Animal Co. Ltd., Shanghai, China) received treatment of saline (n = 10), 15 mg/kg Rb1 (Rb1-L) (n = 10) or 60 mg/kg Rb1 (Rb1-H) (n = 10) in a volume of 100 μL through intraperitoneal injection for 8 weeks. For the experiments involving *Fto* overexpression in endothelial cells *in vivo*, adeno-associated virus serotype 9 (AAV9) carrying *Fto* or AAV9 alone was intravenously delivered to 4-week-old HFD-fed *Apoe* ^−/−^ mice at the dose of 2 × 10^11^ vg per mouse. The experimental groups included the mice transduced with the AAV9 (n = 10), Rb1-treated mice transduced with AAV9 (n = 10) and Rb1-treated mice transduced with AAV9 carrying *Fto* (n = 10). Daily treatment of Rb1 was given at the dose of 60 mg/kg for 4 weeks and AAV9 controls received saline treatment. In some cases, C57BL/6J mice on standard chow diet were analyzed as indicated or subjected to bone marrow isolation and neutrophil enrichment. The experiments were approved by the Institutional Animal Care and Use Committee at Yueyang Hospital of Integrated Traditional Chinese and Western Medicine, Shanghai University of Traditional Chinese Medicine (Approval Numbers: YYLAC-2022-149 and YYLAC-2022-168).

### 2.3 Histological examination and immunohistochemistry

Hematoxylin and eosin (HE) staining, Oil red O staining, and immunohistochemistry were performed on cryosections (8 μm thick) made from aortic roots after fixation in 4% paraformaldehyde. Antibodies used in this work included rabbit anti-CD68 antibody (ab125212, Abcam, United States) (1:500), rabbit anti-VCAM1 antibody (ab134047, Abcam, United States) (1:250) and the donkey anti-rabbit IgG H&L secondary antibody (Alexa Fluor 488) (ab150073, Abcam, United States) (1:1000). The aortic sections were also counter-stained with 4-6-diamidino-2-phenylindole (DAPI) (10236276001, Roche, Switzerland). A light microscopy (DM2000, Leica, Germany) was adopted to acquire micrographs after HE and Oil red O staining. A fluorescent microscope (DM6000B, Leica, Germany) was utilized to record the fluorescent images after immunohistochemical examinations. Image analysis was conducted using Image-Pro Plus 6.0.

### 2.4 Neutrophil isolation and NETs preparation

Bone marrow cells were processed to enrich neutrophils using the Neutrophil Isolation Kit (Solarbio, China). Briefly, red blood cells were lysed before isolation of neutrophils. Isolated neutrophils were cultured in 10-cm cell culture dishes at 5 × 10^5^ cells/mL, followed by exposure to PMA (100 nM) for 4 h. Afterward, the cell culture dishes were thoroughly washed and NETs were collected by vigorous agitation, followed by 5-min centrifugation at 300 × g. The supernatants were collected and subjected to 20-min centrifugation at 12,000 × g at 4 C. After resuspension, the DNA content was estimated using a BioSpec-nano Spectrophotometer (Shimadzu, Japan).

### 2.5 Culture and treatment of endothelial cells

Human aortic endothelial cells (HAECs) (Shanghai Institute of Quicell Biotechnology, China) were cultured in ECM supplemented with reagents from the endothelial cell growth kit (ATCC, United States). HAECs from 2 to 10 passages were used for the experiments. For the immunofluorescence assay, HAECs were plated in 24-wells at 1 × 10^5^ cells/well. For gene expression, dot blotting and Western blotting analyses, HAECs were seeded in 6-well plates at 5 × 10^5^ cells/well. After incubation with saline or Rb1 (100 μM) for 30 min, the cells were exposed to NETs at 1 μg/mL. For the experiments involving *FTO* overexpression, HAECs were plated in 24-well plates at 1 × 10^5^ cells/well. Transfection of *FTO*-overexpressing pcDNA3.1(+) plasmid or pcDNA3.1(+) empty vector was performed using lipofectamine™ 2000 reagent (Invitrogen, United States). Subsequently, 30-min Rb1 (100 μM) or saline incubation was performed, which was followed by 1 μg/mL NETs exposure for 12 h for gene expression analysis or 24 h for adhesion and endothelial barrier function assessments. The dose of Rb1 was selected based on the results from a preliminary study testing the effective dose of Rb1 on neutrophil adhesion to NETs-stimulated endothelial cells, which showed that 100 μM was the optimal dose for inhibiting neutrophil-endothelial cell adhesion in the presence of NETs.

### 2.6 Real-time quantitative polymerase chain reaction (qPCR)

TRIzol reagent (Invitrogen, United States) was used to enrich total RNA from HAECs or mouse aortas. The PrimeScript RT Master Mix (TaKaRa, Japan) was then applied to synthesize cDNA. The LightCycler 480 SYBR Green I Master (Roche, Germany) was used to set up PCR reactions that were analyzed on a LightCycler 480 II system (Roche, United States). The primer sequences were listed in Table 1. Human *GAPDH* or mouse *Gapdh* was examined as internal controls. The fold change analysis of the gene expression was performed according to 2^-[Ct (specific gene)−Ct (*GAPDH*^ ^or^ ^*Gapdh*)]^.

**TABLE 1:** Primer sequences for real-time qPCR analyses.

Gene name	Forward primer	Reverse primer
*ICAM1*	CTC​CAA​TGT​GCC​AGG​CTT​G	CAG​TGG​GAA​AGT​GCC​ATC​CT
*VCAM1*	GAT​GGC​GCC​TAT​ACC​ATC​CG	ATG​ACC​CCT​TCA​TGT​TGG​CTT
*SELE*	CTG​CCA​AGT​GGT​AAA​ATG​TTC​AAG	CTT​GCA​CAC​AGT​GCC​AAA​CAC
*SELP*	TCC​GCT​GCA​TTG​ACT​CTG​GAC​A	CTG​AAA​CGC​TCT​CAA​GGA​TGG​AG
*METTL3*	CTG​CTT​GGT​TGG​TGT​CAA​AGG	GCG​AGT​GCC​AGG​AGA​TAG​TC
*METTL16*	TGG​AGC​AAC​CTT​GAA​TGG​CTG​G	CCA​TCA​GGA​GTG​TCT​TCT​GTG​G
*WTAP*	AAA​GGA​CGG​GGA​GTG​TTA​CC	CAT​CTT​GAA​TCA​GGA​GAG​TCG​C
*YTHDC1*	TCA​GGA​GTT​CGC​CGA​GAT​GTG​T	AGG​ATG​GTG​TGG​AGG​TTG​TTC​C
*YTHDF1*	CAA​GCA​CAC​AAC​CTC​CAT​CTT​CG	GTA​AGA​AAC​TGG​TTC​GCC​CTC​AT
*FTO*	TAC​AAC​GCT​GTC​AGT​TGG​C	GGC​AAG​GAT​GGC​AGT​CAA​GA
*GAPDH*	CCC​GCT​CCC​TCT​TTC​TTT​G	GGG​GCC​ATC​CAC​AGT​CTT​C
*Icam1*	AAA​CCA​GAC​CCT​GGA​ACT​GCA​C	GCC​TGG​CAT​TTC​AGA​GTC​TGC​T
*Vcam1*	GCT​ATG​AGG​ATG​GAA​GAC​TCT​GG	ACT​TGT​GCA​GCC​ACC​TGA​GAT​C
*Sele*	GGA​CAC​CAC​AAA​TCC​CAG​TCT​G	TCG​CAG​GAG​AAC​TCA​CAA​CTG​G
*Selp*	AGC​AGG​GAC​ACT​GAC​AAT​CC	TGT​TCC​TAG​GTG​GCT​GTG​AG
*Gapdh*	CCG​GTG​CTG​AGT​ATG​TCG​TG	CCT​TTT​GGC​TCC​ACC​CTT​C

### 2.7 Cell adhesion assay

HAECs were seeded in 24-well plates at 1 × 10^5^ cells/well. Subsequently, after 30-min treatment of 100 μM Rb1 or saline, the cells were exposed to 1 μg/mL NETs for 24 h. For neutrophil adhesion assay, isolated neutrophils were loaded on the monolayer of HAECs at 2 × 10^4^ per well. After 30-min co-culture, the non-adherent neutrophils were rinsed off. The remaining neutrophils were then stained using anti-Ly6G antibody (127601, BioLegend, United States) (1:1000). For monocyte adhesion assay, THP-1 cells (National Collection of Authenticated Cell Cultures, China) were labeled with CellTracker™ CM-Dil (Invitrogen, United States) (30 min, 37°C). The labeled cells were subsequently loaded on the monolayer of HAECs at 2 × 10^4^ cells/well. After the 30-min co-culture and washing procedures, the fluorescence of the cell tracker was captured using a fluorescence microscope (DMI6000, Leica, Germany) to assess the adherence of THP-1 cells to HAECs. Image-Pro Plus 6.0 was utilized for image analysis.

### 2.8 Endothelial barrier function assays

Twenty-four-well transwell inserts were used for the seeding of HAECs (1 × 10^5^ cells/well). For transendothelial electrical resistance (TEER) assay, 30-min vehicle or Rb1 (100 μM) incubation was followed by 1 μg/mL NETs exposure for 6, 12 and 24 h. A Millicell ERS-2 voltohmmeter (EMD Millipore, Billerica, MA) was used to evaluate TEER. The value was recorded when stable and sustained TEER was established. For Evans blue dye extravasation assay, after 30-min treatment of Rb1 (100 μM) or vehicle, NETs (1 μg/mL) were added and cells were cultured for additional 24 h. Subsequently, the transwell set was washed and the following procedures included adding 600 μL of 4% BSA solution to the lower chamber and 100 μL of 0.67 mg/mL Evans blue dye (in 4% BSA) to the upper chamber. After 1-h equilibration, the upper chambers were discarded. Evans blue dye in the lower chambers was quantified using a microplate reader (Epoch, BioTek, United States) with the absorbance wavelength set at 620 nm.

### 2.9 Dot blotting

Dotting blotting was conducted as detailed in our previous study (Cui et al., 2023). In brief, the miRNeasy Mini Kit (Qiagen, Germany) was used to purify total RNA from HAECs. After denaturation, the RNA was loaded on the nylon membrane (Millipore, United States). The membrane was then UV cross-linked, blocked and sequentially probed with anti-N6-methyladenosine antibody (ab151230, Abcam, United States) (1:2000) and horseradish peroxidase-conjugated goat anti-rabbit secondary antibody (W4011, Promega, United States). The signal was visualized using the Western Bright ECL reagent (Advansta, United States) and recorded using the MiniChemi system (China). Image-Pro Plus 6.0 was adopted for densitometry analysis. RNA loading was estimated by staining the membrane with methylene blue.

### 2.10 Western blotting

RIPA buffer (Beyotime, China) supplemented with phosphatase inhibitors and proteases inhibitors (Roche, United States) was used to prepare protein lysate. The electrophoresis was done in 10% SDS-PAGE gels. The protein was transferred to the polyvinylidene fluoride membrane (Millipore, United States). The membrane was probed with rabbit anti-FTO primary antibody (ab124892, Abcam, United States) (1:1000) or mouse anti-β-actin primary antibody (66009, Proteintech, China) (1:3000) at 4°C overnight, followed by secondary antibody incubation using horseradish peroxidase-conjugated goat anti-rabbit antibody (W4011, Promega, United States) (1:3000) or goat anti-mouse secondary antibody (W4021, Promega, United States) (1:3000). SuperSignal™ West Pico PLUS Chemiluminescent Substrate (Thermo Fisher Scientific, United States) was used to visualize the signal. Images were obtained using the MiniChemi system (China). Densitometry was assessed by Image-Pro Plus 6.0.

### 2.11 Statistical analysis

The data were shown as mean ± standard error of the mean (SEM). SPSS 26.0 software (Ehningen, Germany) or Graphpad Prism 9 software (San Diego, United States) was employed for statistical analysis. Normality test and homogeneity of variance were assessed by Shapiro-Wilk test and Levene’s test, respectively. Multiple comparisons were made by one-way ANOVA with Tukey’s HSD *post hoc* analysis. Statistically significance was defined as P < 0.05.

## 3 Results

### 3.1 Rb1 attenuates endothelial activation and atherosclerosis *in vivo*

**FIGURE 1:**
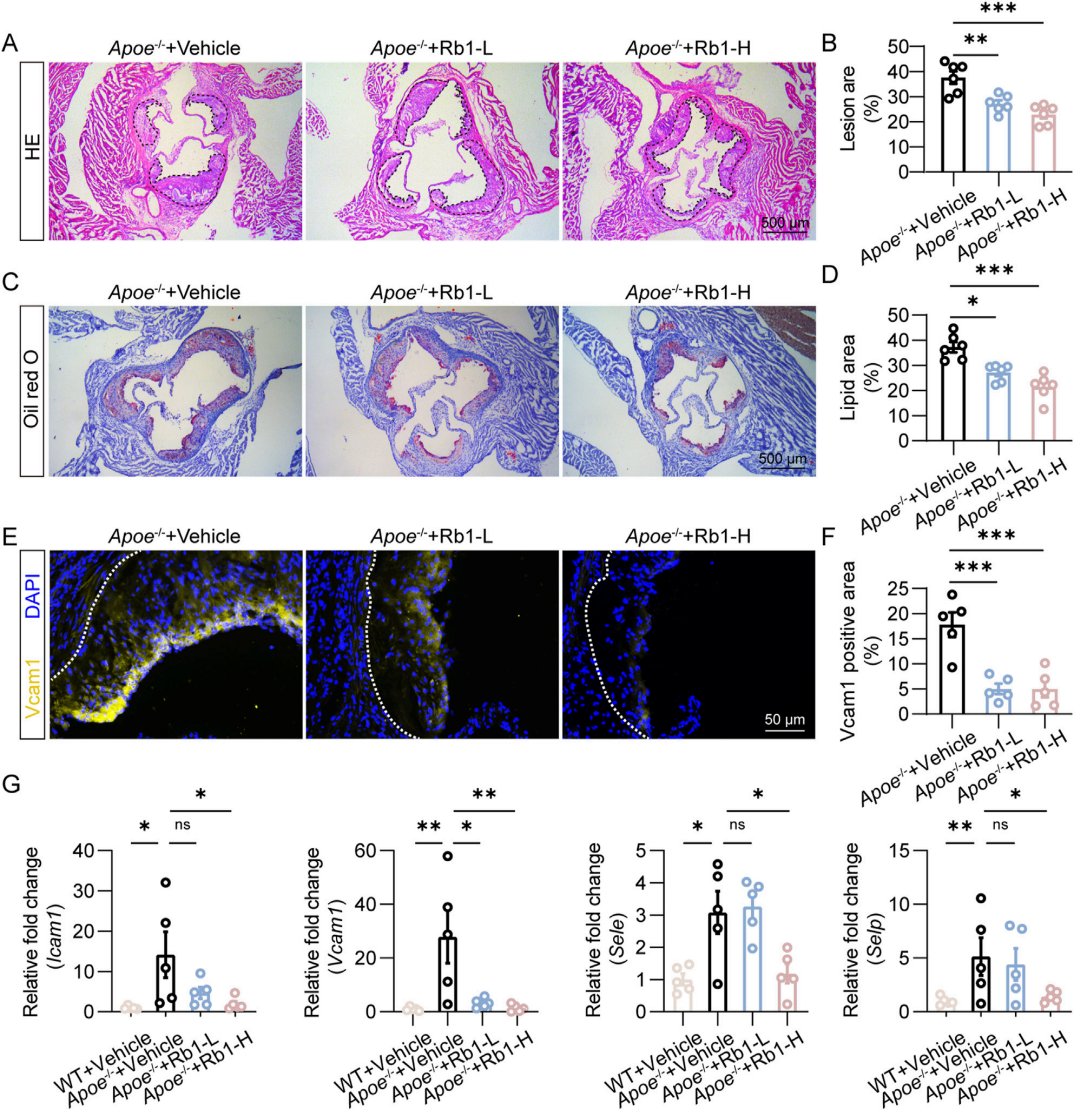
Rb1 mitigates atherosclerosis and endothelial activation in HFD-fed *Apoe* ^−/−^ mice. **(A)** Representative microscopic images of HE-stained aortic root cross-sections. Scale bar, 500 μm. **(B)** The area of the atherosclerotic lesion normalized against the lumen area (%) (n = 6 per group). **(C)** Oil red O positivity in the aortic root cross-sections. Scale bar, 500 μm. **(D)** Quantification of the plaque lipid load (n = 6 per group). **(E)** Vcam1 immunopositivity (in yellow) associated with the atherosclerotic lesions. DAPI highlighted the nuclei (in blue). Scale bar, 50 μm. **(F)** Quantification of Vcam1 immunopositivity in the atherosclerotic lesions (n = 5 per group). **(G)** The aortic expression of *Icam1*, *Vcam1*, *Sele* and *Selp*. Relative fold change was plotted against the wild type controls (n = 5 per group). *P < 0.05, **P < 0.01, ***P < 0.001, ns, not significant.

Prior to investigate the direct impact of Rb1 on NETs-induced endothelial activation, we evaluated the pharmacological effects of Rb1 treatment on atherosclerosis-associated endothelial activation in the HFD-fed *Apoe* ^−/−^ mice *in vivo*. Rb1 treatment resulted in smaller area of the atherosclerotic lesion (Figures 1A,B), which was corroborated by reductions in the lipid load at lesion sites as revealed by Oil red O staining (Figures 1C,D). Furthermore, the immunopositivity of Vcam1 in the atherosclerotic plaques was remarkably decreased due to Rb1 treatment (Figures 1E,F). The vehicle-treated HFD-fed *Apoe* ^*−/−*^ mice were characterized by increased aortic expression of the genes encoding Icam1, Vcam1, E-selectin and P-selectin. However, decreased aortic expression of Icam1, Vcam1, E-selectin and P-selectin was noted as a result of Rb1 treatment (Figure 1G). These results support that Rb1 treatment mitigates endothelial activation and atherosclerosis *in vivo*.

### 3.2 Rb1 mitigates NETs-induced aberrant activation of endothelial cells

**FIGURE 2:**
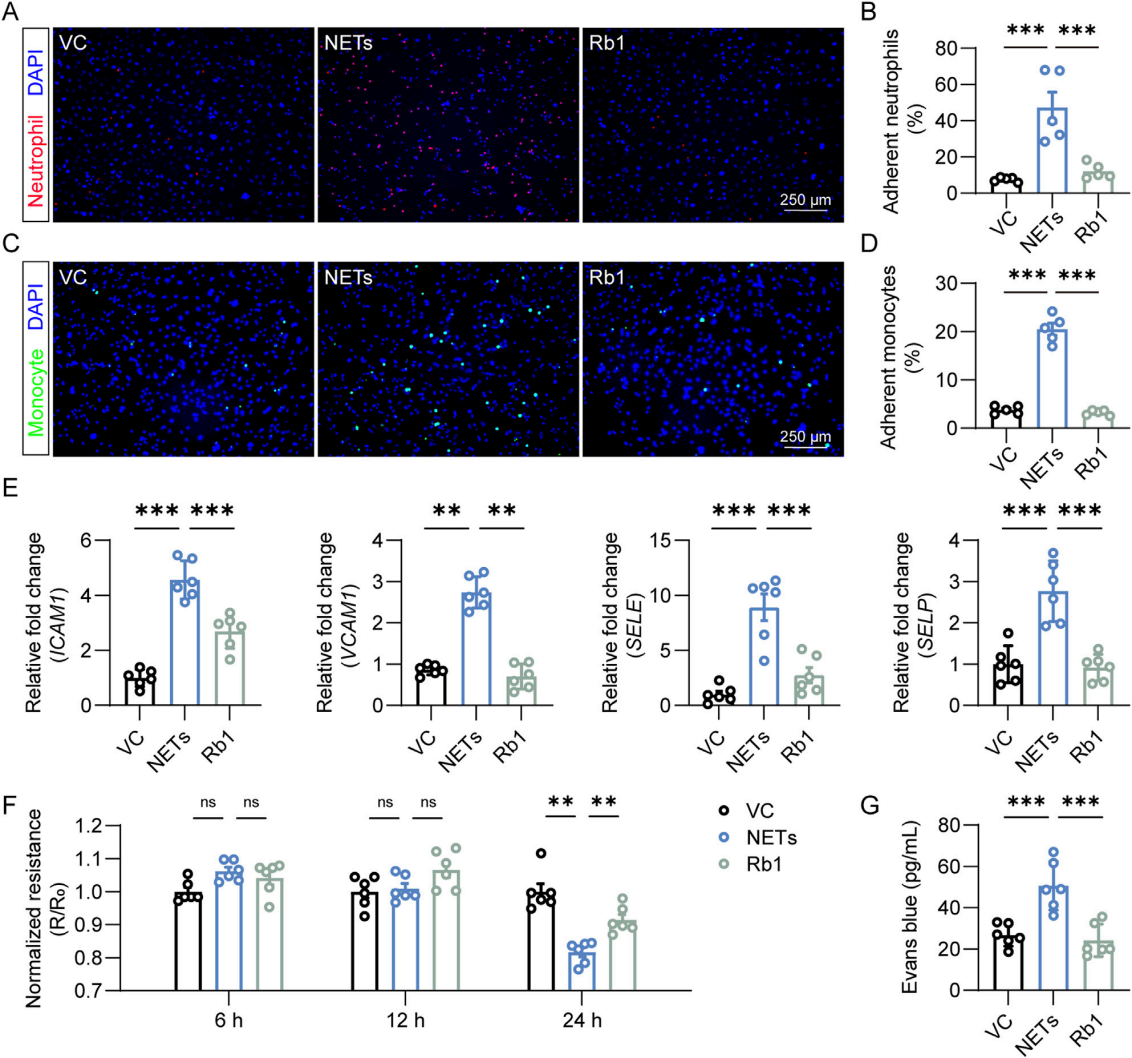
Rb1 inhibits NETs-stimulated activation of endothelial cells. **(A)** Adhesion of neutrophils (in red) to HAECs (nuclei were counterstained with DAPI, in blue). Scale bar, 250 μm. **(B)** Proportion of neutrophils adhering to HAECs (n = 5 per group). **(C)** Adhesion of THP-1 monocytes (in green) to HAECs (nuclei were counterstained with DAPI, in blue). Scale bar, 250 μm. **(D)** Proportion of monocytes adhering to endothelial cells (n = 5 per group). **(E)** The expression of *ICAM1*, *VCAM1*, *SELE* and *SELP* in HAECs. Relative fold change was plotted against VC (n = 6 per group). **(F)** TEER assay. Resistance (R) was normalized against the starting resistance (R_0_) (n = 6 per group). **(G)** Evans blue extravasation assay. The amount of Evans blue dye detected in the lower chamber of the transwell (n = 6 per group). **P < 0.01, ***P < 0.001, ns, not significant.

Next, we evaluated the pharmacological effect of Rb1 on NETs-stimulated endothelial activation *in vitro*. Activated endothelial cells are characterized by augmented expression of genes that encode adhesion molecules, which facilitate the capture of leukocytes, an essential step for the subsequent transendothelial leukocyte migration. Consistent with this notion, NETs exposure led to increased expression of *ICAM1*, *VCAM1*, *SELE* and *SELP* in endothelial cells. In contrast, much lower expression of *ICAM1*, *VCAM1*, *SELE* and *SELP* was found in the Rb1-treated cells (Figure 2A). In agreement with these results, augmented adhesion of neutrophils occurred as a result of NETs stimulation. However, much lower level of neutrophil adhesion was noted in the NETs-stimulated endothelial cells treated with Rb1 (Figures 2B,C). Similar observations were also made when THP-1 monocytes were loaded on the endothelial cells (Figures 2D,E). Additionally, prolonged stimulation of NETs decreased the TEER value, whereas much higher TEER value was documented in the Rb1-treated NETs-stimulated cells (Figure 2F). Similar observations were made when extravasation of Evans blue dye across the endothelial cells was measured (Figure 2G). Taken together, these results indicate that Rb1 mitigates NETs-induced aberrant activation of endothelial cells.

### 3.3 Rb1 treatment augments m^6^A RNA modification in NETs-stimulated endothelial cells

**FIGURE 3:**
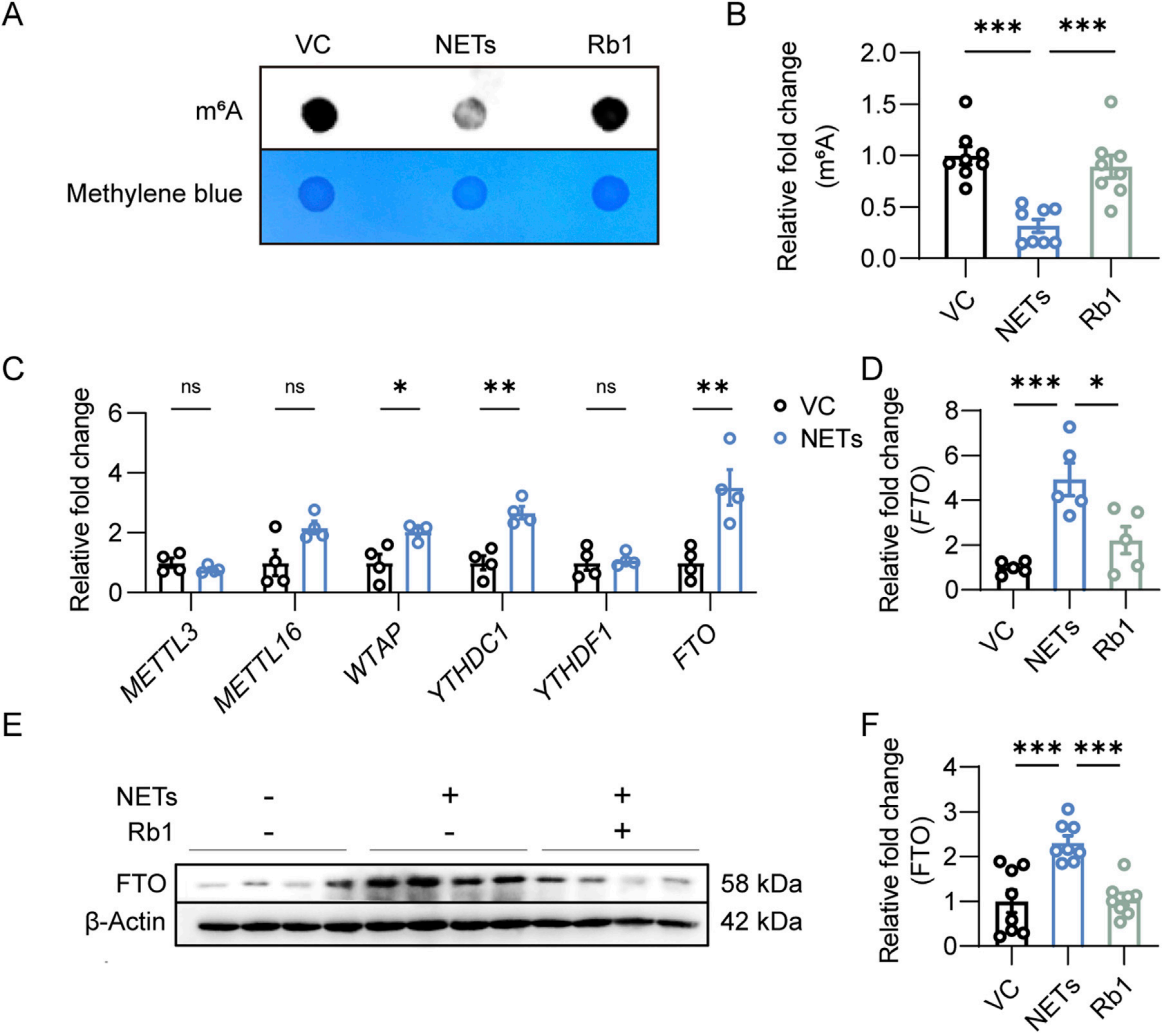
Rb1 increases m^6^A RNA methylation and decreases the expression of FTO in the NETs-stimulated endothelial cells. **(A)** Dot blotting analysis of m^6^A RNA methylation in HAECs. **(B)** Relative fold change in the level of m^6^A RNA modification (n = 8 per group). **(C)** Real-time qPCR analysis of the expression of *METTL3*, *METTL16*, *WTAP*, *YTHDC1*, *YTHDF1* and *FTO* in HAECs. Relative fold change was plotted against VC (n = 4 per group). **(D)** The expression of *FTO* in HAECs. Relative fold change was plotted against VC (n = 5 per group). **(E)** Western blotting analysis of the protein expression of FTO in HAECs. **(F)** Relative fold change was plotted against VC (n = 8 per group). *P < 0.05, **P < 0.01, ***P < 0.001, ns, not significant.

Furthermore, m^6^A RNA methylation was evaluated to probe the mechanistic implications of m^6^A RNA modification in NETs-induced endothelial activation. NETs stimulation led to overt reductions in m^6^A RNA methylation in endothelial cells. On the contrary, significantly higher level of m^6^A RNA methylation was observed in the NETs-stimulated cells treated with Rb1 (Figures 3A,B). To understand the mechanisms underlying altered m^6^A RNA methylation in NETs-stimulated endothelial cells, the expression of RNA methyltransferases such as *METTL3*, *METTL16* and *WTAP*, readers of m^6^A on RNA such as *YTHDC1* and *YTHDF1* as well as RNA demethylase *FTO* was assessed. Increased expression of *WTAP*, *YTHDC1* and *FTO* was observed in the NETs-stimulated endothelial cells (Figure 3C). Given that NETs stimulation led to lower level of m^6^A RNA modification in endothelial cells (Figures 3A,B), we focused on FTO in the context of NETs-induced endothelial activation. As shown in Figure 3D, in contrast to NETs-induced upregulation of *FTO* expression, Rb1 treatment led to significantly lower endothelial expression of *FTO* in the presence of NETs. Consistently, the protein level of FTO was increased in response to NETs stimulation, whereas significantly decreased level of FTO protein was noted in the Rb1-treated cells (Figures 3E,F). Collectively, these results suggest the possibility that Rb1 may increase the level of m^6^A RNA methylation by decreasing the expression of FTO in NETs-stimulated endothelial cells, an effect contributing to Rb1-conferred attenuation of NETs-induced endothelial activation.

### 3.4 Rb1 suppresses NETs-induced endothelial activation in part by lowering the expression of FTO in endothelial cell*s*

**FIGURE 4:**
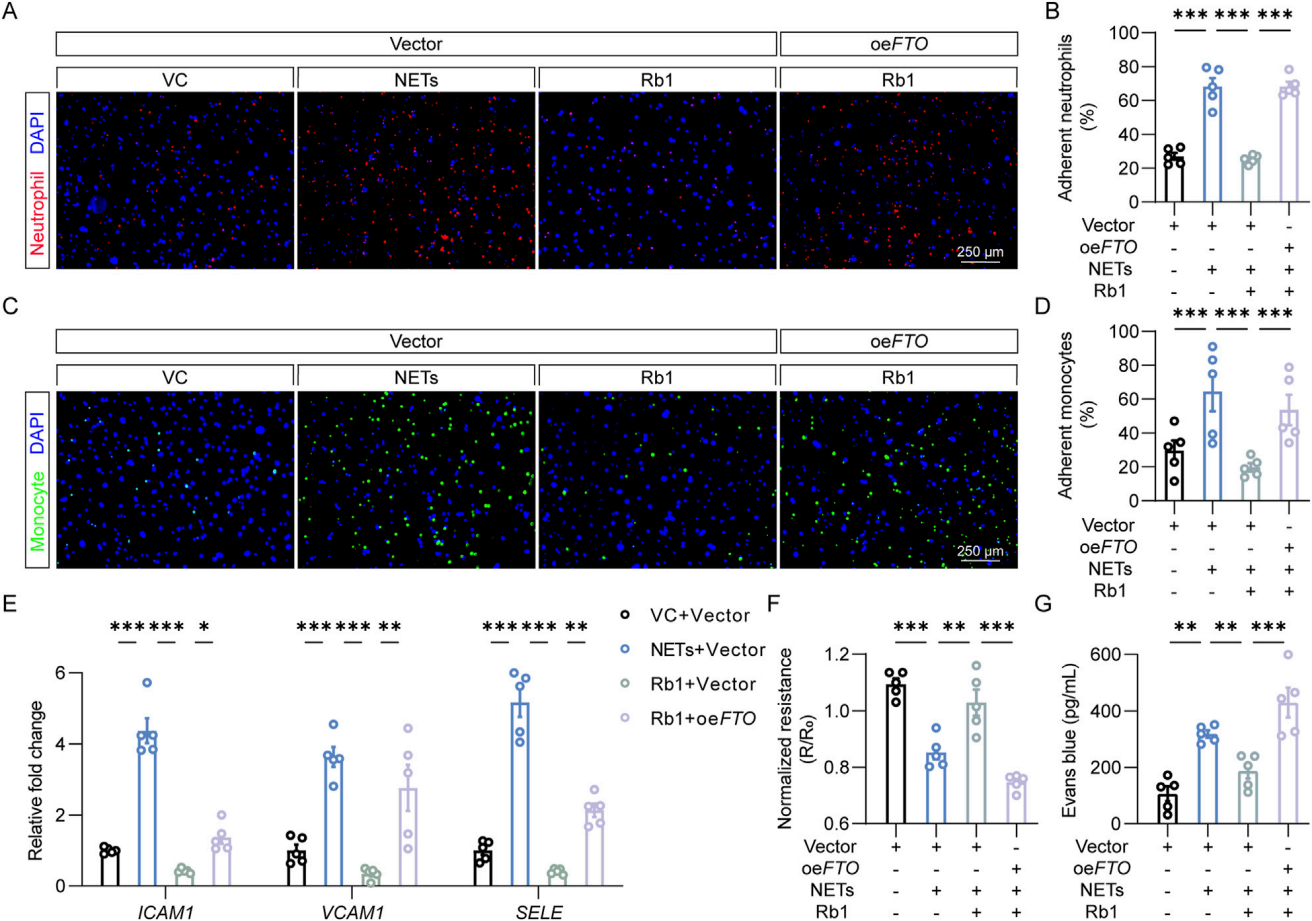
*FTO* overexpression abolishes the effects of Rb1 against NETs-stimulated endothelial activation. **(A)** Adhesion of neutrophils (in red) to HAECs (DAPI counterstaining of the nuclei, in blue). Scale bar, 250 μm. **(B)** Proportion of neutrophils adhering to HAECs (n = 5 per group). **(C)** Adhesion of monocytes (in green) to HAECs (DAPI counterstaining of the nuclei, in blue). Scale bar, 250 μm. **(D)** Proportion of monocytes adhering to endothelial cells (n = 5 per group). **(E)** The expression of *ICAM1*, *VCAM1* and *SELE* in HAECs. Relative fold change was plotted against VC (n = 5 per group). **(F)** TEER in HAECs. Resistance (R) was normalized against the starting resistance (R_0_) (n = 5 per group). **(G)** Evans blue dye extravasation assay. The amount of Evans blue dye detected in the lower chamber of the transwell (n = 5 per group). *P < 0.05, **P < 0.01, ***P < 0.001.

To address the possibility that FTO is mechanistically implicated in Rb1-conferred protection against NETs-induced endothelial activation, the adhesion of neutrophils or monocytes to endothelial cells was assessed after transfecting HACEs with empty vector or *FTO-*overexpressing plasmid. As shown in Figures 4A,B, attenuated neutrophil-endothelial adhesion was consistently observed in the Rb1-treated NETs-exposed cells. However, *FTO* overexpression significantly diminished the beneficial effects of Rb1 against NETs-enhanced neutrophil adhesion to the endothelial cells. Similar observations were made when adhesion of THP-1 monocytes to endothelial cells was examined (Figures 4C,D). In agreement, *FTO* overexpression partially abolished the inhibitory effects of Rb1 on NETs-induced expression of *ICAM1*, *VCAM1* and *SELE* (Figure 4E). Furthermore, *FTO* overexpression decreased TEER values and increased Evans blue dye extravasation in the Rb1-treated NETs-stimulated HAECs (Figures 4F,G). Altogether, the results here validate the hypothesis that Rb1 protects against NETs-stimulated endothelial activation in part by decreasing the expression FTO in endothelial cells.

### 3.5 Fto overexpression in endothelial cells abolishes the anti-atherosclerotic effects of Rb1 *in vivo*

**FIGURE 5:**
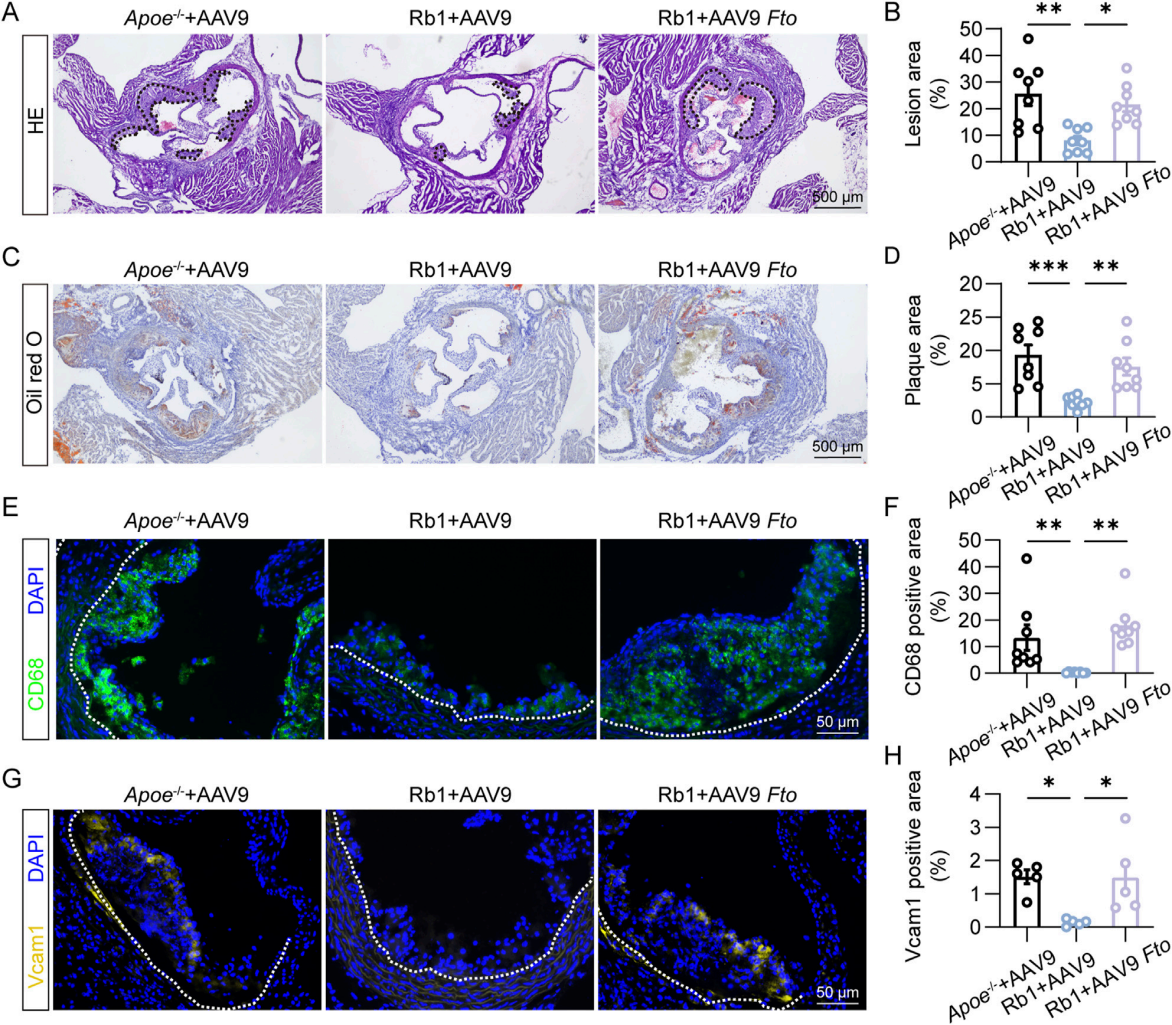
*Fto* overexpression offsets Rb1-conferred protection against atherosclerosis and endothelial activation in HFD-fed *Apoe* ^−/−^ mice. **(A)** Representative microscopic images of HE-stained aortic root cross-sections. Scale bar, 500 μm. **(B)** The area of the atherosclerotic lesion normalized against the lumen area (%) (n = 8 per group). **(C)** Oil red O positivity in the atherosclerotic plaques. Scale bar, 500 μm. **(D)** Quantification of the Oil red O-positive lipid load in the plaques (n = 8 per group). **(E)** CD68 immunopositivity (in green) and DAPI counterstaining of the nuclei (in blue) in the plaques. Scale bar, 50 μm. **(F)** Quantification of CD68 immunopositivity in the atherosclerotic lesions (n = 8 per group). **(G)** Vcam1 immunopositivity (in yellow) and DAPI counterstaining of the nuclei (in blue) in the plaques. Scale bar, 50 μm. **(H)** Quantification of Vcam1 immunopositivity (n = 5 per group). *P < 0.05, **P < 0.01, ***P < 0.001.

To further address the mechanistic implications of FTO in Rb1-conferred protection against aberrant endothelial activation in the pathological context of atherosclerosis *in vivo*, AAV9-mediated overexpression of *Fto* was targeted to the endothelial cells via a Tie2 promoter and delivered to the HFD-fed *Apoe* ^*−/−*^ mice in conjunction with Rb1 treatment. As shown in Figures 5A,B, smaller atherosclerotic lesions were consistently observed in the Rb1-treated HFD-fed *Apoe* ^*−/−*^ mice transduced with AAV9 control, whereas significantly increased lesion size was readily detected in the Rb1-treated HFD-fed *Apoe* ^*−/−*^ mice transduced with *Fto*-overexpressing AAV9. Meanwhile, increased lipid load (Figures 5C,D) and macrophage burden (Figures 5E,F) were noted in the Rb1-treated HFD-fed *Apoe* ^*−/−*^ mice transduced with *Fto*-overexpressing AAV9. Lastly, the *in situ* expression of Vcam1 in the atherosclerotic plaques was significantly higher in the Rb1-treated HFD-fed *Apoe* ^*−/−*^ mice transduced with *Fto*-overexpressing AAV9 (Figures 5G,H). These results indicate that *Fto* overexpression in endothelial cells abolishes the endothelial protective and anti-atherosclerotic effects of Rb1 *in vivo*.

## 4 Discussion

**FIGURE 6:**
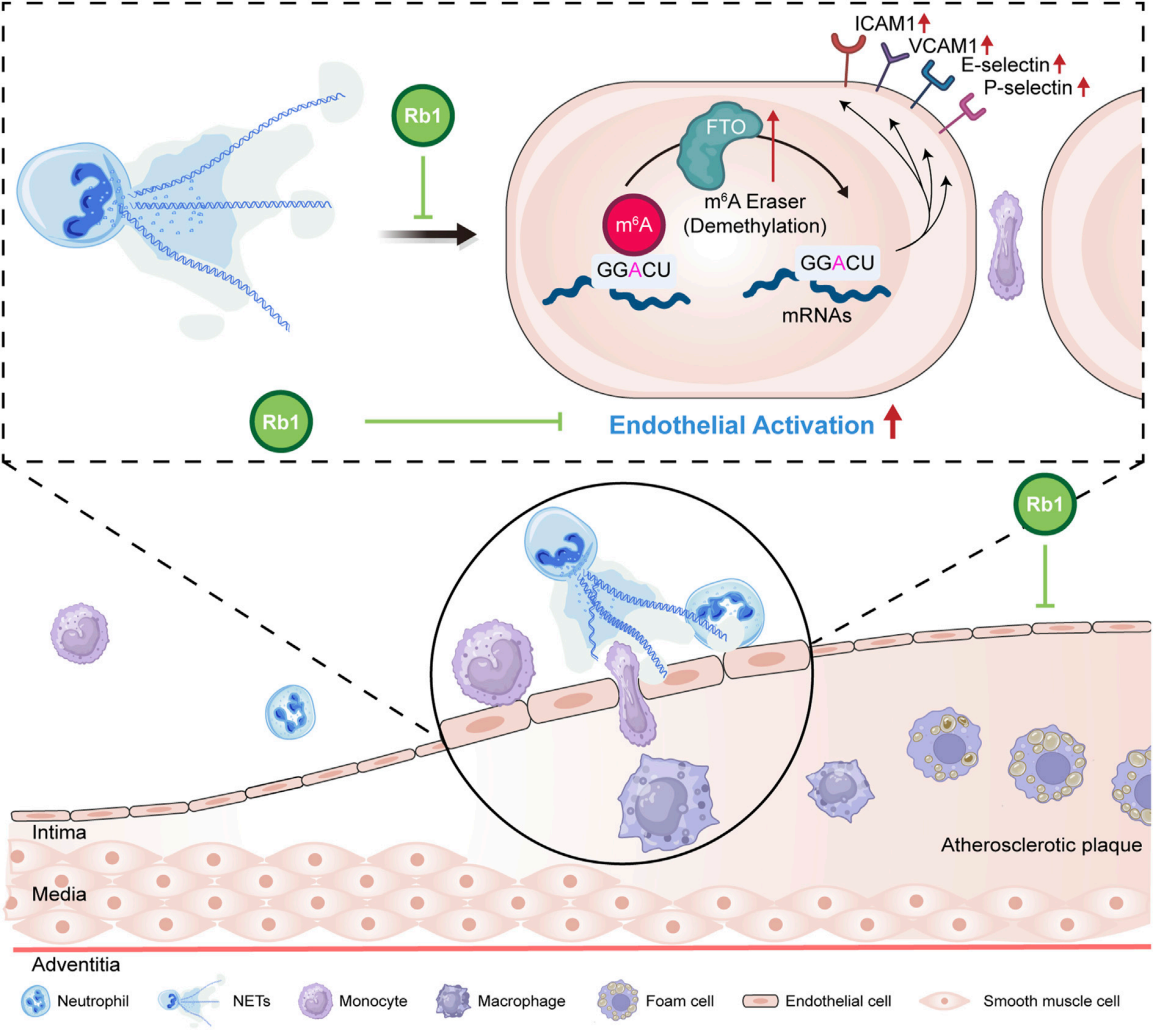
A schematic illustration of the pharmacological effects of Rb1 against NETs-induced endothelial activation in atherosclerosis. Our work here reveals that Rb1 mitigates NETs-stimulated aberrant activation of endothelial cells. Furthermore, Rb1 counteracts NETs-triggered dysregulation of FTO and m^6^A methylation in endothelial cells, which may in part contribute to the pharmacological effects of Rb1 against atherosclerosis.

Atherosclerosis is essentially an inflammatory disorder in part driven by the interplay between vascular endothelium and immune cells. Accumulating evidence supports the mechanistic contributions of neutrophil-derived NETs in the pathogenesis of atherosclerosis. Here we demonstrate that the anti-atherosclerotic effects of Rb1 involve mitigating NETs-stimulated endothelial activation, which is in part mediated by offsetting FTO-mediated m^6^A demethylation in endothelial cells (Figure 6).

First, our current study reveals that Rb1 mitigates NETs-stimulated aberrant activation of endothelial cells. Atherosclerosis is initiated by insults to the vascular endothelium. The ensuing endothelial activation promotes aberrant leukocyte-endothelium interactions that occur through the course of atherosclerosis. This process is tightly regulated by adhesion molecules (e.g., ICAM, VCAM, E-selectin and P-selectin) that undergo upregulated expression during atherosclerosis (Richardson et al., 1994; Poston et al., 1992; Johnson-Tidey et al., 1994; Nakashima et al., 1998; Li et al., 1993). NETs cause direct damage to endothelial cells (Gupta et al., 2010; Villanueva et al., 2011). Consistent with this notion, our study uncovers that NETs upregulate the expression of the genes encoding vascular adhesion molecules and enhances the adhesion of neutrophils and monocytes to endothelial cells, supporting the direct implications of NETs in endothelial activation. Furthermore, our findings uncover that Rb1 mitigates NETs-induced expression of pro-atherogenic adhesion molecules (*ICAM1*, *VCAM1*, *SELE*, and *SELP*) as well as the adhesion of neutrophils and monocytes to endothelial cells. ICAM1 mediates firm adhesion of leukocytes to the endothelial cells that undergo activation (Collins et al., 2000). VCAM1 preferentially mediates the recruitment of mononuclear leukocytes (Nakashima et al., 1998; Cybulsky and Gimbrone, 1991; Sakai et al., 1997). The selectins determine the tethering, rolling and adhesion of all types of leukocytes that interact with the activated endothelium (Collins et al., 2000; Dong et al., 1998). Therefore, the cellular and molecule evidence of Rb1-conferred protection against NETs-stimulated aberrant interaction between neutrophils/monocytes and endothelial cells provides new insights into the mechanisms underlying the anti-atherosclerotic effects of Rb1. Notably, the NETs-modulating and endothelial protective effects have also been demonstrated in a study elucidating the anti-atherosclerotic mechanisms of a traditional Chinese medicine formula comprising *Prunus persica* (L.) Batsch, *Carthamus tinctorius* L., *Angelica sinensis* (Oliv.) Diels, *Conioselinum anthriscoides* ‘Chuanxiong’, *Paeonia lactiflora* Pall., *Rehmannia glutinosa* (Gaertn.) Libosch. ex DC., *Astragalus mongholicus* Bunge, *Cinnamomum verum* J.Presl, and *Epimedium sagittatum* (Siebold & Zucc.) Maxim. (Shao et al., 2024). These findings, in conjunction with our current data, provide experimental evidence supporting the concept that polyherbal preparations, botanical drugs, and plant metabolites warrant further investigation for their potential pharmacological effects in mitigating atherosclerotic progression through modulation of NETs-induced endothelial activation.

Secondly, our findings reveal that FTO-mediated m^6^A RNA demethylation is in part implicated in the pharmacological effects of Rb1 against NETs-induced endothelial activation in atherosclerosis. The level of m^6^A RNA methylation is decreased in the human atherosclerotic plaques (Quiles-Jiménez et al., 2020). Importantly, FTO expression can be induced by oxidized LDL (ox-LDL). Overexpression of *FTO* results in increased expression of ICAM1 and VCAM1. On the contrary, *FTO* knockdown attenuates ox-LDL-induced or TNFα-triggered expression of ICAM1 and VCAM1 in endothelial cells, leading to reduced adhesion of monocytes to activated endothelial cells (Mo et al., 2022). FTO-mediated RNA demethylation also drives diabetes-associated endothelial activation (Zhou et al., 2023). These findings support the notion that FTO plays an important role in endothelial activation. Moreover, NETs-m^6^A crosstalk has been documented in the pathological context of lung cancer, pulmonary injury, and glioma (Xu et al., 2025; Zhang et al., 2022; Dai et al., 2024). Our results here further demonstrate that m^6^A RNA methylation is markedly reduced in the NETs-stimulated endothelial cells, which is in part explained by significantly increased expression of FTO. Therefore, our findings not only corroborate the known implications of FTO in endothelial activation and NETs-m^6^A crosstalk, but also add new mechanistic understanding of endothelial activation by linking NETs to FTO-regulated m^6^A modification in endothelial activation. Furthermore, Rb1 treatment results in higher levels of m^6^A RNA methylation and decreased expression of FTO in the NETs-stimulated endothelial cells. Forced expression of *FTO* in part cancels out Rb1-conferred protection against NETs-induced endothelial activation *in vitro*. Most importantly, *Fto* overexpression partially offsets the anti-atherosclerotic effects of Rb1 in the HFD-fed *Apoe* ^−/−^ mice *in vivo*. Therefore, the molecular, cellular and *in vivo* evidence here jointly indicate that Rb1 increases the level of m^6^A RNA methylation in the NETs-stimulated endothelial cells in part by decreasing the expression of FTO, which contributes to the effects of Rb1 at attenuating endothelial activation and progression of atherosclerotic lesions. However, the in-depth mechanisms underlying the regulated expression of FTO in response to Rb1 treatment are to be further illustrated in the future. Meanwhile, further studies are necessary to identify specific downstream m^6^A targets that are modified by Rb1 treatment in the NETs-stimulated endothelial cells in the context of atherosclerosis.

Moreover, it is worth noting that our findings here are in line with the research advances on the context-dependent impact of plant metabolites on m^6^A RNA modification. To name a few, rhein has been uncovered to be a substrate competitive inhibitor of FTO and this FTO inhibitory activity of rhein contributes to its anti-cancer effects (Chen et al., 2012; Yang et al., 2021). Baicalin has recently been revealed to mitigate male reproductive toxicity by restoring the expression of m^6^A reader YTHDC2 (Wen et al., 2025). Baicalin has also been reported to alleviate lactate-induced acidification in tumor microenvironment by decreasing the level of m^6^A reader IGF2BP3 in oral squamous cell carcinoma cells (Cui et al., 2025). Our previous work has also shown that the expression of FTO can be increased by cinnamic acid in cardiomyocytes, which in part accounts for cinnamic acid-conferred protection against pressure overload-induced left ventricular hypertrophy (Cui et al., 2023). Therefore, our current findings add to the growing body of evidence that supports the pharmacological potentials of plant metabolites on m^6^A RNA modification.

In conclusion, the present study reveals that Rb1 mitigates NETs-induced aberrant endothelial activation in atherosclerosis. Furthermore, the newly identified pharmacological effects of Rb1 against NETs-induced endothelial activation in part involve augmenting the level of m^6^A RNA methylation by suppressing FTO expression in endothelial cells. The experimental evidence presented in this work thus provides new mechanistic insights into the anti-atherosclerotic effects of Rb1.

## Data Availability

The original contributions presented in the study are included in the article/supplementary material, further inquiries can be directed to the corresponding authors.
